# Antimicrobial and antioxidant activities of fennel oil

**DOI:** 10.6026/97320630018795

**Published:** 2022-09-30

**Authors:** Sheeba Naaz, Nadeem Ahmad, M. Irfan Qureshi, Nadeem Hashmi, Mohd Sayeed Akhtar, M. Masroor A. Khan

**Affiliations:** 1 Department of Biotechnology, Jamia Millia Islamia, New Delhi - 110025, India; 2 Department of Biosciences, Jamia Millia Islamia, New Delhi - 110025, India; 3 Department of Botany, Aligarh Muslim University, Aligarh - 202002, India; 4 Department of Botany, Gandhi Faiz-e-Aam College, UP - 242001, India

**Keywords:** Foeniculum vulgare, antibacterial, antioxidant activity, essential oil composition, GC, DPPH

## Abstract

It is of interest to estimate the effects of foliar spray (ISA) on essential oil yield, chemical constituents, antioxidant, and antimicrobial activities of fennel (Foeniculum vulgare Mill).Fennel was treated with ISA solutions at 40 and 80 mg L-1 doses. Application of ISA significantly augmented antioxidant and antimicrobial activities in addition to essential oil yield and its principal elements in fennel. 80 mg L−1 dose of ISA was found to be pre-eminent. Antioxidant properties of EOs were determined through DPPH assays, metal chelators and lipid peroxidation. While antimicrobial activities were evaluated using agar well diffusion and microdilution techniques of broth. Gram positive and Gram negative bacteria were used to gauge the oil's antibacterial effectiveness. Data shows that antioxidant and antimicrobial activities of fennel oil were found to be the highest. According to GC analysis, trans-anethole (78.38-86.08%), methyl chavicol (2.32-2.54%), and fenchone (6.65-8.95%) were the three main constituents of fennel essential oil.

## Background:

Background: Essential oils are intricate volatile molecules found in plant parts and comprise several kinds of molecules i.e terpenoids, terpenes, phenols, aromatic and aliphatic constituents [1]. They are good choice for pharmaceuticals and flavoring intent. Their popularity increases as they inhibited the growth of microbes and extended the life of foodstuffs as compared to synthetic preservatives which are expensive and customers don't rely on them [1]. Sodium alginate (SA) is a natural polysaccharide. Gamma-rays irradiation degraded SA into small oligomers with a little molecular weight (MW). When plants are foliar sprayed by these oligomers, they aggravate several biological and physiological activities, including elevating the growth of plants, germination of seeds, elongation of shoots, growth of roots, production of flowers, mitigation of toxic metals etc [2, 3, 4, 5, 6]. Fennel (Foeniculum vulgare Mill, Family-Apiaceae) is an essential aromatic plant with medicinal properties and has well-defined anti-inflammatory and antimicrobial activities [7]. It has traditionally been regarded as a spice and a medicinal herb. It is a highly valued medicinal crop that is used as an anti-inflammatory, anti-oxidant, intoxicant, gastrointestinal, mucolytic, and spasmolytic agent [6, 7]. Fennel essential oil is important to the pharmaceutical industries as well as other segments [6, 8, 9]. It is known that trans-anethole, methyl-chavicol and fenchone are essential constituents of fennel seed oil [6, 10, 11]. However, there is less information available on the antioxidant, antimicrobial, activities, and chemical composition of essential oil of fennel. Therefore, it is of interest to explore the essential oils' active constituents and antioxidant capacity of fennel seed. Keeping the pharmaceutical worth of fennel in mind, current research was conducted to see whether the application of degraded sodium alginate via gamma rays can enhance the phytochemical characteristics, antioxidant and antimicrobial activities of fennel EO.

## Methodology:

### Seed sowing:

Viable seeds of fennel were bought from CIMAP, Lucknow, UP, India. The seeds were decontaminated with ethanol (95 %). Each earthen pot held five kg of a uniform blend of organic manure and soil in 1:4 ratio. Before seed sowing, a homogeneous dose of 25-25-15 kg ha-1 of a base fertilizer including potassium, nitrogen and phosphorus was applied.

### Sodium alginate irradiation:

Gamma rays raised from the Co-60 source were used to irradiate Sodium Alginateat BARC, Mumbai (Figure 1 - see PDF). DDW was used to prepare various application of ISA 0, 40, and 80 mg L−1 for foliar sprays.

### Experimental setup and treatments:

The experiment was carried out in the green house of the Botany Department, AMU, Aligarh, India. The pots were placed in a randomized block pattern. VariousISA applications [0, 40, and 80 mgL-1] were used as treatments. The ISA treatments were given to plants as foliar sprays at 10 days after sowing (DAS) intermission.

### Gas chromatography (GC) / Essential oil (EO) composition:

The active compositions of EO (Fenchone, methyl chavicol, and trans-anethole) were analyzed using the GC equipped with an integrator, flame ionization detector, and SE-30 stainless steel column. As the carrier gas, nitrogen was employed. The GC was run at the following temperatures: 250°C; for the detector, 160°C; for the oven, and 250°C; for the injector. The active ingredients were identified based on retention duration, and their quantity was determined by evaluating the experimental data peaks to those acquired from the published standard value [11, 12].

### Antioxidant activity:

#### DPPH radical-scavenging activity:

DPPH was assessed according to Mahakunakorn [13]. Three concentrations of EO i.e 20, 40 and 80 µg/mL in MeOH with DPPH of 0.1mM were produced. At 517 nm, scavenging activity was detected spectrophotometrically. Asc (Vit C) was taken as control. Percentage inhibition plot was made alongside sample concentration. The subsequent equation was used to compute the DPPH Inhibition (%):

% Inhibition = [A0–A1] X 100 / A0

Where A = absorbance 

### Ferrous ion chelating ability assay (FIC):

The method by Singh and Rajini is applied for FIC assessment [14]. The spectro-photometric measurement of the solutions was taken at 562 nm absorbance. The following formula used to calculate the % FIC,

% Inhibition = [A0–A1] X 100 / A1

Where A = absorbance

### Thiobarbituric Acid Reactive Species test (TBARS):

Lipid peroxidation (TBARS), was assessed through the technique of Daker [15]. The taken standards were BHT and ascorbic acid. The following formula was used to compute the inhibition (%):

% Inhibition = [A0–A1]X 100 / A0

Where A = absorbance

### Antimicrobial activity:

#### Microorganisms:

For the antibacterial assessment of the fennel's oils, four bacterial species - S. aureus, P. aeruginosa, B. Subtilis and E. coli were taken. These bacteria were obtained from HiMedia Lab Pvt. Ltd, India. Disc diffusion technique was utilized to evaluate the antibacterial properties of essential oils. Using ampicillin as an antibacterial standard and positive control, the experiment was performed. By computing the sizes of the inhibitory zones (measured in milli meters) around each disc, the antibacterial activities were evaluated.

### Statistical analysis:

All data were taken together for variance analysis (ANOVA) to identify differences (p< 0.05). The means were compared using Tukey's test in order to regulate whether there were any substantial alterations in the levels of the primary component.

## Results and Discussion:

### The yield of essential oil and chemical compounds

Fennel oil's GC chromatograms were compared to those that were previously used as a reference. Principal constituents of fennel EO were expressly accelerated by ISA, according to the outcomes of GC studies. The chief chemical compositions of fennel EO were trans-anethole (82.43-86.68), fenchone (6.65-8.95%), and methyl chavicol (2.32-2.54%) based on their tR, KI, and mass spectral data, the active ingredients of fennel oil were identified and the total ion chromatograms of fennel oil shown in (Figure 2-3 - see PDF). Among different concentrations, 80 mg L−1 of ISA proved to be the best. ISA80 significantly enhanced the yield and main components of essential oil of fennel (Table 1 - see PDF). Fennel essential oil mostly contains anethole, methyl chavicol and fenchone [6,16]. The ISA treatment has been demonstrated to be positively helpful in increasing the growth, yield, and EO content of Menthaar vensis, a type of medicinal and aromatic plant [17]. Augmentation in yield of essential oil and its chief ingredients resulted from the ISA- accelerated growth, nutrient (N and P) buildup and leaf oil gland's population, they all contributed to an increase in essential oil output and its main constituents [2, 6]. As a result of ISA's favorable impact on plant metabolism and on the enzymes involved in mono- or sesquiterpene-biosynthesis, which might have been exploited to speed up the production of metabolites relevant to oil generation? The outcome on fennel is compatible with these results [5, 6, 8, 9, 18].

### Antioxidant activity:

Numerous studies have shown that phytochemicals, from various sources, revealed potent antioxidant activity [10, 19]. The antioxidant activity of EO was calculated thru DPPH free radical scavenging, the TBARS and the (FIC) assay. Ascorbic acid and BHT were employed as positive controls. These tests were performed at varying concentrations of essential oils i.e, 20, 40, and 80 mg/mL. The values of concentrations that subdued IC50 values (50 % in each test) are shown in Table 2(see PDF). Dose of 80 mg L-1 of ISA gives the best results which was greater than ascorbic acid or BHT, Fennel exhibited the strongest antioxidant potential in satisfying the DPPH radical. Parallel results were obtained in thyme [20]. If the EO comprises a lot of phenolic components, a good antioxidant property is anticipated [21]. Thus, fenchone and anethole, which inhibit oxidation, is related to the higher phenylic acid and antioxidant activity of the fennel examined in this study. Our results are in agreement with those of Ruberto and Baratta [22] who stated that hydrocarbons of monoterpenes, which are EO components of plants such as thyme and oregano, exhibited a noteworthy influence in the inhibition of oxidation-peroxidation. Similar findings were also attained from the FRAP assay, ISA80 significantly increased antioxidant activity in comparison to ascorbic acid and BHT. In the TBARS, the EO of fennel was revealed to demonstrate inhibitory efficacy against lipid peroxidation (Table 2 - see PDF). The fact that the Fe2+ complex formation had not surfaced in the occurrence of essential oils of fennel which recommends that the Fe is chelated by the essential oil. The scavenging consequence of fennel oils and standards on metals abridged. The information reported in Table 2 shows that the fennel has an extraordinary capability for Fe-binding, which acted as an oxidation protector that was related to its Fe-binding capability. The results of this investigation specify that fennel essential oil has the capacity to act as a free radical inhibitor. Both the primary antioxidant activity and the scavenging activity react to free radicals, which may reduce the amount of oxidative injury that occurs inside humans.

### Antimicrobial activity:

The antimicrobial effect of fennel EO has been observed against gram (-) and gram (+) bacteria and their capacity were assessed by the minimum inhibition concentrations and inhibition zone diameters (Table 3 - see PDF). For the assessment of antibacterial activity, ampicillin was taken as a reference material. According to the findings, fennel EO was proved to be more effective antibacterial than the standard commercial Ampicillin. Fennel EO was less effective than ampicillin in the bioassays for P. aeruginosa and E. coli, respectively (Table 3 - see PDF), however it proved to be the best essential oil for fighting Gram-positive bacteria which provided an inhibitory zone that is wider than ampicillin's in the tests for B. subtilis and S. aureus, respectively (Table 3 - see PDF). These outcomes are parallel with [23, 24] who described that, the essential oil's antibacterial activity was found to be quite strong. According to Khaldun, who claimed that fennel oil had a strong anti-bacterial impact on Candida albicans (fungal strain), our observed actions are consistent with his findings [25].

## Conclusions:

The chief constituents of fennel’s oil displayed higher antioxidant and antibacterial activities, the main composition of fennel oil were trans-anethole (82.43-86.68%), fenchone (6.65–8.95%), and methyl chavicol (2.32-2.54%). Fennel essential oil exhibited a potential source of antioxidants while it proved to be the most effective against Gram (+) bacteria. The application of irradiated sodium alginate resulted in enhancement of the main constituents’ i.e trans-anethole, methyl chavicol, and fenchone, and improvement in the yield of fennel EO with 80 mg L−1 proved to be the prominent ISA treatment (Figure 4 - see PDF). Such ISA-dependent improvement in antimicrobial and antioxidant activity of fennel EO, was figured out first time in the course of this research. Moreover, the action mechanism of EOs against antimicrobial activity is still unclear, so further studies are needed to illustrate the action mechanism.
